# Development of a Multiplex PCR Assay for Selected *Escherichia coli* Virulence Genes, *Clostridium perfringens cpa* and *Cryptosporidium* 18S rRNA in Faecal Samples from Diarrheic Dairy Calves

**DOI:** 10.3390/biology15120921

**Published:** 2026-06-12

**Authors:** Jian-Jun Hou, Jia-Qi Zhao, Ying-Ying Fan, Ming-Yi Zhang, Jun-Ke Song, Xin Yang, Guang-Hui Zhao

**Affiliations:** College of Veterinary Medicine, Northwest A&F University, Xianyang 712000, China; 13709853911@163.com (J.-J.H.); 2410779848@nwafu.edu.cn (J.-Q.Z.); yingyingfan@nwafu.edu.cn (Y.-Y.F.); 2019010830@nwafu.edu.cn (M.-Y.Z.); sjk7998@163.com (J.-K.S.)

**Keywords:** *Escherichia coli*, *Clostridium perfringens*, *Cryptosporidium*, multiplex PCR, dairy calves

## Abstract

*Escherichia coli*, *Clostridium perfringens* and *Cryptosporidium* are common diarrhea-related pathogens in dairy calves, posing considerable economic losses to animal husbandry and threatening public health. A previous study in our lab found the frequent occurrence of *Escherichia coli* virulence genes (*eaeA*, *stx1* and *stx2*), *Clostridium perfringens cpa* and *Cryptosporidium* in diarrheic dairy calves in Ningxia Hui Autonomous Region, China. The present study aimed to develop a multiplex PCR for simultaneous detection of these virulence genes and *Cryptosporidium* in diarrheic dairy calves. The multiplex PCR demonstrated sensitivities of 2060 copies, 18200 copies, 1300 copies, 1990 copies and 974 copies for *stx1*, *stx2*, *eaeA*, *cpa* and *18S rRNA*, respectively. Moreover, the method showed no cross-reactivity with *Giardia duodenalis*, *Enterocytozoon bieneusi*, *Eimeria*, *Haemonchus contortus*, *Oesophagostomum*, *Moniezia*, *Salmonella*, *Proteus mirabilis* and *Staphylococcus aureus*. Further application of the multiplex PCR in 20 clinical faecal samples from diarrheic dairy calves found that the positive rates of the multiplex PCR assay were 55% (11/20), 50% (10/20), 60% (12/20), 45% (9/20) and 25% (5/20) for *stx1*, *stx2*, *eaeA*, *cpa* and *18S rRNA*, respectively, which were not significantly lower than those of the conventional PCR targeting *stx1* (60%, 12/20) and *eaeA* (65%, 13/20), but higher than those of the reported PCR targeting *stx2* (45%, 9/20) and *cpa* (40%, 8/20), and were consistent with those of the reported nested PCR targeting *18S rRNA* (25%, 5/20). Taken together, the present study preliminarily developed a multiplex PCR assay for the rapid detection of selected *E. coli* virulence genes, *C. perfringens cpa* and *Cryptosporidium 18S rRNA* in dairy calves, which could provide basic data and technical support for the diagnosis and prevention of calf diarrhea. However, more samples from divergent clinical settings are needed to validate the assay in the diagnosis of selected *E. coli* virulence genes, *C. perfringens cpa* and *Cryptosporidium 18S rRNA* in future studies.

## Simple Summary

Diarrhea is a significant disease in dairy calves, with pathogen infection being one of the primary causes. Our previous study found the frequent occurrence of *Escherichia coli* virulence genes (*eaeA*, *stx1* and *stx2*), *Clostridium perfringens cpa* and *Cryptosporidium* in diarrheic dairy calves in Ningxia Hui Autonomous Region, China. The present study aimed to develop a multiplex PCR assay for the simultaneous detection of selected *E. coli* virulence genes, *C. perfringens cpa* and *Cryptosporidium 18S rRNA* in diarrheic dairy calves, which could contribute to the prevention and control of diarrheic diseases in dairy calves.

## 1. Introduction

Diarrhea is one of the commonest diseases in dairy calves on large-scale farms and also a major cause of increased calf mortality and economic losses for farms [1]. Calves are highly susceptible to diarrhea within the first month after birth, particularly during the second to third week [2]. Acute diarrhea can cause fever, rapid breathing, and reduced appetite in affected calves, ultimately leading to death from dehydration and exhaustion. Chronic or persistent diarrhea may stunt growth, delay the time to maturity, reduce the productive and reproductive performance of cows, and significantly lower the economic returns of farms [3].

Calf diarrhea is a complex multifactorial disease, and its occurrence is attributed to various factors, including both infectious and non-infectious elements [4,5]. Infection with pathogens such as bacteria, viruses, and parasites represents one of the primary causes [4,6]. Among these infectious pathogens, the main bacteria include *Escherichia coli*, *Salmonella* and *Clostridium perfringens* [7,8,9]; the primary viruses include bovine coronavirus (BCV), bovine rotavirus (BRV) and bovine viral diarrhea virus (BVDV) [10,11,12]; and the parasites include *Cryptosporidium*, *Giardia duodenalis* and *Enterocytozoon bieneusi* [13,14,15]. Understanding the occurrence and distribution of these diarrhea-related pathogens is essential for the prevention and control of calf diarrhea.

Accurate diagnosis facilitates understanding of pathogen epidemiology. Using PCR-based sequencing techniques, our previous study identified a high prevalence of *E. coli* virulence genes (*stx1*, *stx2* and *eaeA*), *C. perfringens cpa* and *Cryptosporidium 18S rRNA* in diarrheic calves in Ningxia Hui Autonomous Region, China [16]. In the present study, we developed a multiplex PCR assay capable of simultaneously detecting these virulence genes and *Cryptosporidium 18S rRNA* gene, aiming to provide a rapid, economical and efficient clinical diagnostic tool for detecting intestinal pathogens in dairy calves with diarrhea. This established multiplex PCR method will therefore help farms implement timely and effective prevention and control measures, thereby reducing the occurrence and transmission of calf diarrhea.

## 2. Materials and Methods

### 2.1. Genomic DNA Isolation

Genomic DNA was extracted from clinical faecal samples of diarrheic calves using the E.Z.N.A. Stool DNA kit (Omega, Norcross, GA, USA) according to the manufacturer’s instructions. Genomic DNA samples of *E. coli*, *C. perfringens*, *Cryptosporidium*, *Giardia duodenalis*, *Enterocytozoon bieneusi*, *Eimeria*, *Haemonchus contortus*, *Oesophagostomum*, *Moniezia*, *Salmonella*, *Proteus mirabilis* and *Staphylococcus aureus* were obtained from a previous study in our laboratory. All genomic DNA samples were stored at −20 °C.

### 2.2. Construction of Recombinant Plasmids

*E. coli* virulence genes (*eaeA*, *stx1* and *stx2*), *C. perfringens cpa* and *Cryptosporidium 18S rRNA* were amplified from the genomic DNA by PCR as previously described [17,18,19]. The sequences of the *E. coli* virulence genes (*eaeA*, *stx1* and *stx2*), *C. perfringens cpa* and *Cryptosporidium 18S rRNA* cloned into plasmids in this study are provided in Table S1. A universal DNA Purification Kit (Tiangen, Beijing, China) was used to recover the target gene fragments, and the gel extraction products were ligated by using the pMD 19-T Vector Cloning Kit (Takara, Dalian, China). The ligation products were then transformed into *E. coli* JM109 competent cells. After verification by PCR-based sequencing, the positive mono-colonies were further cultured for plasmid DNA extraction using the TIANpure Midi Plasmid Kit (Tiangen, Beijing, China). The concentration of the extracted plasmid DNA was measured using a spectrophotometer (Allsheng, Hangzhou, China), and the DNA was stored at −20 °C until use.

### 2.3. Primer Design

Primers targeting the selected *E. coli* virulence genes (*eaeA*, *stx1* and *stx2*), *C. perfringens cpa* and *Cryptosporidium 18S rRNA* were designed using Primer Premier 6 (PREMIER Biosoft, San Francisco, CA, USA) and synthesized by Sangon Biotech (Shanghai, China) (Table 1).

### 2.4. Establishment of Gene-Specific PCRs for Detecting Selected E. coli Virulence Genes, C. perfringens cpa and Cryptosporidium 18S rRNA

Each gene-specific PCR was performed by using a PCR machine (SimpliAmp^TM^, Thermo Fisher Scientific, Waltham, MA, USA) with a temperature ramp rate of 1 °C/s. The reaction mixture consisted of a total volume of 25 μL containing 1.0 μL of DNA template, 0.125 μL of *Ex Taq* polymerase, 2.5 μL of 10 × *Ex Taq* Buffer (Mg^2+^ free), 2.0 μL of MgCl_2_ (25 mM), 2.0 μL of dNTP (2.5 mM each), 1.0 μL each of forward and reverse primers (10 μM), and 15.375 μL of ddH_2_O under the following conditions: initial denaturation at 94 °C for 5 min, followed by 35 cycles of 94 °C for 30 s, 55 °C for 30 s and 72 °C for 1 min, and a final extension at 72 °C for 10 min.

Subsequently, the reaction conditions and reaction system of each gene-specific PCR were optimized to improve PCR efficiency. Firstly, the annealing temperature was optimized over a range of 53 °C to 60 °C at 1 °C intervals. Next, primer concentrations were optimized from 0.16 μM to 0.72 μM in 0.08 μM increments. Finally, the Mg^2+^ concentration was optimized from 0.5 mM to 4.0 mM in 0.5 mM increments.

To assess the sensitivity, positive plasmid DNAs containing the selected *E. coli* virulence genes, *C. perfringens cpa* and *Cryptosporidium 18S rRNA* were subjected to tenfold serial dilutions. Using the optimal reaction conditions and system established in this study, the lowest copy number of each plasmid detectable by its gene-specific PCR assay was determined.

Using the optimal reaction conditions and system established in this study, positive DNA samples were used as controls. To evaluate the specificity of each gene-specific PCR assay, DNA samples from *G. duodenalis*, *E. bieneusi*, *Eimeria*, *H. contortus*, *Oesophagostomum*, *Moniezia*, *Salmonella*, *P. mirabilis* and *S. aureus* were also included.

### 2.5. Establishment of a Multiplex PCR for Simultaneously Detecting Selected E. coli Virulence Genes, C. perfringens cpa and Cryptosporidium 18S rRNA

Based on the optimal reaction conditions and systems established for the gene-specific PCR targeting *E. coli stx1*, specific primers for *E. coli stx2* and *eaeA*, *C. perfringens cpa* and *Cryptosporidium* 18S rRNA were sequentially added to the PCR reaction mixture to perform multiplex PCR amplification. Through gradual optimization of the annealing temperature and primer concentrations, and Mg^2+^ concentrations, a multiplex PCR assay was successfully established.

A PCR reaction system with a total volume of 25 μL was used. The initial reaction mixture consisted of 1.0 μL of DNA template, 0.125 μL of *Ex Taq*, 2.5 μL of 10 × *Ex Taq* Buffer (Mg^2+^ free), 2.0 μL of MgCl_2_ (25 mM), 2.0 μL of dNTP (2.5 mM each), 0.8 μL of each of the upstream and downstream primers (10 μM) for *E. coli stx1*, and 0.6 μL each of the upstream and downstream primers for *E. coli stx2*, and the remaining volume was made up of ddH_2_O. The PCR amplification conditions were as follows: initial denaturation at 94 °C for 5 min; 35 cycles of 94 °C for 30 s, 55 °C for 30 s, and 72 °C for 1 min; and a final extension at 72 °C for 10 min.

#### 2.5.1. Optimization of the Multiplex PCR for Detecting the *stx1* and *stx2* Genes of *E. coli*

The annealing temperature for the multiple PCR was optimized over a range of 53 °C to 60 °C at 1 °C intervals. Subsequently, the concentration of the upstream and downstream primers for *E. coli stx2* was optimized by adding them to the PCR reaction system at final concentrations ranging from 0.16 μM to 0.72 μM in 0.08 μM increments.

#### 2.5.2. Optimization of the Multiplex PCR for Detecting the *stx1*, *stx2* and *eaeA* Genes of *E. coli*

Using the optimal reaction conditions and systems established for the multiple PCR targeting *E. coli stx1* and *stx2*, the annealing temperature was optimized over a range of 53 °C to 60 °C at 1 °C intervals. Subsequently, the primer concentration for *E. coli eaeA* was optimized by adding upstream and downstream primers to the reaction system at final concentrations ranging from 0.16 μM to 0.72 μM in 0.08 μM increments.

#### 2.5.3. Optimization of the Multiplex PCR for Detecting Selected *E. coli* Virulence Genes and *C. perfringens cpa*

Based on the optimal reaction conditions and systems established for the multiplex PCR targeting *E. coli stx1*, *stx2* and *eaeA*, the annealing temperature was optimized over a range of 53 °C to 60 °C at 1 °C intervals. Subsequently, gradient concentrations of the upstream and downstream primers for *C. perfringens cpa* were tested by adding them to the PCR reaction system at final concentrations ranging from 0.16 μM to 0.72 μM in 0.08 μM increments.

#### 2.5.4. Optimization of the Multiplex PCR for Detecting Selected *E. coli* Virulence Genes, *C. perfringens cpa* and *Cryptosporidium 18S rRNA*

Using the optimal reaction conditions and systems established for the multiplex PCR targeting the selected *E. coli* virulence genes and *C. perfringens cpa*, the annealing temperature was further optimized over a range of 53 °C to 60 °C at 1 °C intervals. Subsequently, the primer concentration for *Cryptosporidium 18S rRNA* was optimized by adding upstream and downstream primers at final primer concentrations ranging from 0.16 μM to 0.72 μM in 0.08 μM increments. Based on the optimized multiplex PCR system, the Mg^2+^ concentration was then optimized over a final concentration ranging from 0.5 mM to 4.0 mM in 0.5 mM increments to determine the optimal Mg^2+^ concentration for the multiplex PCR.

#### 2.5.5. Sensitivity Test

The positive plasmid DNA templates for *E. coli eaeA*, *stx1* and *stx2*, *C. perfringens cpa* and *Cryptosporidium 18S rRNA* were each subjected to tenfold serial dilutions. Using the optimal reaction conditions and systems established in this study, the lowest copy number of each plasmid detectable by the multiplex PCR assay was determined.

#### 2.5.6. Specificity Test

Using the optimal reaction conditions and systems established in this study, DNA samples positive for *E. coli*, *C. perfringens* and *Cryptosporidium* were used as controls, and DNA samples from *G. duodenalis*, *E. bieneusi*, *Eimeria*, *H. contortus*, *Oesophagostomum*, *Moniezia*, *Salmonella*, *P. mirabilis* and *S. aureus* were included to evaluate the specificity of the multiplex PCR assay.

#### 2.5.7. Clinical Sample Test

The multiplex PCR assay established in this study was used to detect 20 faecal samples randomly collected from diarrheic calves on large-scale farms in Lingwu, Ningxia. The results were compared with those obtained from previously reported gene-specific PCR assays to preliminarily evaluate the applicability of the established method.

#### 2.5.8. Statistical Analysis

Differences in the positive rates of the targeted genes between the multiplex PCR and gene-specific PCR were analyzed by using the chi-square test in SPSS V18.0 (IBM, New York, NY, USA). Differences were considered statistically significant when the *p*-value was less than 0.05.

## 3. Results

### 3.1. Establishment of Gene-Specific PCRs

#### 3.1.1. Optimal Reaction Conditions and Reaction System for the Gene-Specific PCR Targeting *E. coli eaeA*

**Figure 1:**
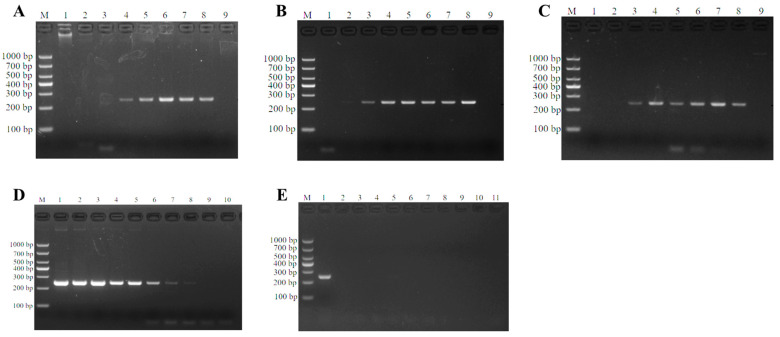
Establishment of the specific PCR assay targeting *E. coli eaeA*. (**A**) Optimization of annealing temperature: M, DL1000 Marker; lanes 1–8, annealing temperature of 53.0 °C, 54.0 °C, 55.0 °C, 56.0 °C, 57.0 °C, 58.0 °C, 59.0 °C and 60.0 °C, respectively; lane 9, negative control. (**B**) Optimization of primer concentration: M, DL1000 Marker; lanes 1–8, primer concentrations of 0.16 μM, 0.24 μM, 0.32 μM, 0.40 μM, 0.48 μM, 0.56 μM, 0.64 μM, and 0.72 μM, respectively; lane 9, negative control. (**C**) Optimization of MgCl_2_ concentration: M, DL1000 Marker; lanes 1–8, MgCl_2_ concentrations of 0.5 mM, 1.0 mM, 1.5 mM, 2.0 mM, 2.5 mM, 3.0 mM, 3.5 mM, and 4.0 mM, respectively; lane 9, negative control. (**D**) Sensitivity test: M, DL2000 DNA Marker; lane 1, 1.30 × 10^9^ copies; lane 2, 1.30 × 10^8^ copies; lane 3, 1.30 × 10^7^ copies; lane 4, 1.30 × 10^6^ copies; lane 5, 1.30 × 10^5^ copies; lane 6, 1.30 × 10^4^ copies; lane 7, 1.30 × 10^3^ copies; lane 8, 1.30 × 10^2^ copies; lane 9, 1.30 × 10^1^ copies; lane 10, negative control. (**E**) Specificity test: M, DNA Marker DL1000; lane 1, *E. coli stx1*-positive strain; lane 2, *Giardia duodenalis*; lane 3, *Eimeria*; lane 4, *Moniezia*; lane 5, *Enterocytozoon bieneusi*; lane 6, *Haemonchus contortus*; lane 7, *Oesophagostomum*; lane 8, *Salmonella*; lane 9, *Proteus mirabilis*; lane 10, *Staphylococcus aureus*; lane 11, negative control.

The optimal annealing temperature for the gene-specific PCR targeting *E. coli eaeA* was 58 °C (Figure 1A); the optimal primer concentration was 0.72 μM (Figure 1B); and the optimal Mg^2+^ concentration was 3.5 mM (Figure 1C). The final optimal PCR reaction system consisted of 1.0 μL of template DNA, 0.125 μL of *Ex Taq*, 2.5 μL of 10 × *Ex Taq* Buffer (Mg^2+^ free), 3.5 μL of MgCl_2_ (25 mM), 2 μL of dNTP (2.5 mM each), and 1.8 μL each of the upstream and downstream primers (10 μM) for *E. coli eaeA*, with the remaining volume made up of ddH_2_O. The PCR conditions were as follows: initial denaturation at 94 °C for 5 min; 35 cycles of 94 °C for 30 s, 58 °C for 30 s, and 72 °C for 1 min; and a final extension at 72 °C for 10 min.

The original concentration of the positive plasmid pMD19-T-*eaeA* extracted in this study was 1.30 × 10^9^ copies/μL. The sensitivity test showed that the minimum detection limit of the gene-specific PCR targeting *E. coli eaeA* was 130 copies (Figure 1D). The specificity test demonstrated that this PCR method amplified the expected band of 248 bp for *E. coli eaeA*, while no amplification was observed for DNA from other common enteric microorganisms (Figure 1E).

#### 3.1.2. Optimal Reaction Conditions and Reaction System for the Gene-Specific PCR Targeting *E. coli stx1*

**Figure 2:**
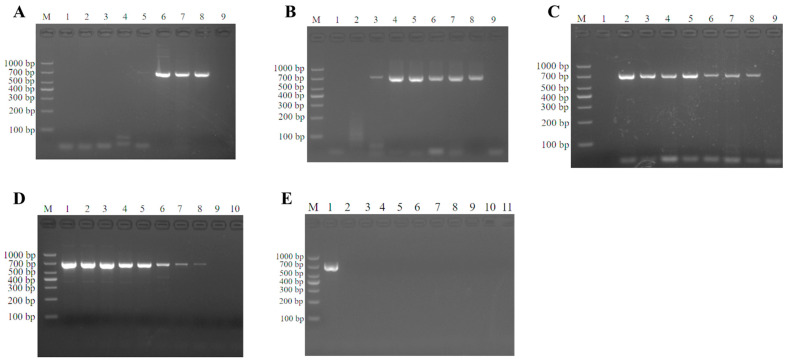
Establishment of the specific PCR assay targeting *E. coli stx1*. (**A**) Optimization of annealing temperature: M, DL1000 Marker; lanes 1–8, annealing temperature of 53.0 °C, 54.0 °C, 55.0 °C, 56.0 °C, 57.0 °C, 58.0 °C, 59.0 °C and 60.0 °C, respectively; lane 9, negative control. (**B**) Optimization of primer concentration: M, DL1000 Marker; lanes 1–8, primer concentrations of 0.16 μM, 0.24 μM, 0.32 μM, 0.40 μM, 0.48 μM, 0.56 μM, 0.64 μM, and 0.72 μM, respectively; lane 9, negative control. (**C**) Optimization of MgCl_2_ concentration: M, DL1000 Marker; lanes 1–8, MgCl_2_ concentrations of 0.5 mM, 1.0 mM, 1.5 mM, 2.0 mM, 2.5 mM, 3.0 mM, 3.5 mM, and 4.0 mM, respectively; lane 9, negative control. (**D**) Sensitivity test: M, DL2000 DNA Marker; lane 1, 2.06 × 10^9^ copies; lane 2, 2.06 × 10^8^ copies; lane 3, 2.06 × 10^7^ copies; lane 4, 2.06 × 10^6^ copies; lane 5, 2.06 × 10^5^ copies; lane 6, 2.06 × 10^4^ copies; lane 7, 2.06 × 10^3^ copies; lane 8, 2.06 × 10^2^ copies; lane 9, 2.06 × 10^1^ copies; lane 10, negative control. (**E**) Specificity test: M, DNA Marker DL1000; lane 1, *E. coli stx1*-positive strain; lane 2, *G. duodenalis*; lane 3, *Eimeria*; lane 4, *Moniezia*; lane 5, *E. bieneusi*; lane 6, *H. contortus*; lane 7, *Oesophagostomum*; lane 8, *Salmonella*; lane 9, *P. mirabilis*; lane 10, *S. aureus*; lane 11, negative control.

The optimal annealing temperature for the gene-specific PCR targeting *E. coli stx1* was 58 °C (Figure 2A); the optimal primer concentration was 0.40 μM (Figure 2B); and the optimal Mg^2+^ concentration was 1.0 mM (Figure 2C). The final established PCR reaction system consisted of 1.0 μL of template DNA, 0.125 μL of *Ex Taq*, 2.5 μL of 10 × *Ex Taq* Buffer (Mg^2+^ free), 1.5 μL of MgCl_2_ (25 mM), 2 μL of dNTP (2.5 mM each), and 1.0 μL each of the upstream and downstream primers (10 μM) for *E. coli stx1*, with the remaining volume made up of ddH_2_O. The annealing temperature was set at 58 °C, and the other reaction conditions were the same as those described for the gene-specific PCR targeting *E. coli eaeA*.

The original concentration of the positive plasmid pMD19-T-*stx1* extracted in this study was 2.06 × 10^9^ copies/μL. The sensitivity test showed that the detection limit of the gene-specific PCR targeting *E. coli stx1* was 206 copies (Figure 2D). The specificity test showed that this PCR method amplified the target band of 706 bp for *E. coli stx1*, while no amplification was observed for DNA from other common enteric microorganisms (Figure 2E).

#### 3.1.3. Optimal Reaction Conditions and Reaction System for the Gene-Specific PCR Targeting *E. coli stx2*

**Figure 3:**
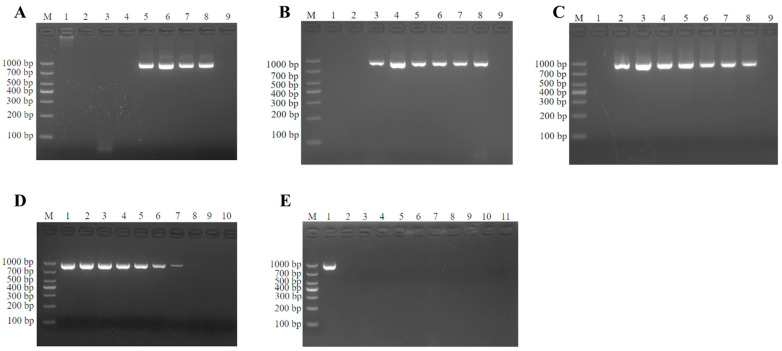
Establishment of the specific PCR assay targeting *E. coli stx2*. (**A**) Optimization of annealing temperature: M, DL1000 Marker; lanes 1–8, annealing temperature of 53.0 °C, 54.0 °C, 55.0 °C, 56.0 °C, 57.0 °C, 58.0 °C, 59.0 °C and 60.0 °C, respectively; lane 9, negative control. (**B**) Optimization of primer concentration: M, DL1000 Marker; lanes 1–8, primer concentrations of 0.16 μM, 0.24 μM, 0.32 μM, 0.40 μM, 0.48 μM, 0.56 μM, 0.64 μM, and 0.72 μM, respectively; lane 9, negative control. (**C**) Optimization of MgCl_2_ concentration: M, DL1000 Marker; lanes 1–8, MgCl_2_ concentrations of 0.5 mM, 1.0 mM, 1.5 mM, 2.0 mM, 2.5 mM, 3.0 mM, 3.5 mM, and 4.0 mM, respectively; lane 9, negative control. (**D**) Sensitivity test: M, DL2000 DNA Marker; lane 1, 1.82 × 10^9^ copies; lane 2, 1.82 × 10^8^ copies; lane 3, 1.82 × 10^7^ copies; lane 4, 1.82 × 10^6^ copies; lane 5, 1.82 × 10^5^ copies; lane 6, 1.82 × 10^4^ copies; lane 7, 1.82 × 10^3^ copies; lane 8, 1.82 × 10^2^ copies; lane 9, 1.82 × 10^1^ copies; lane 10, negative control. (**E**) Specificity test: M, DNA Marker DL1000; lane 1, *E. coli stx2*-positive strain; lane 2, *G. duodenalis*; lane 3, *Eimeria*; lane 4, *Moniezia*; lane 5, *E. bieneusi*; lane 6, *H. contortus*; lane 7, *Oesophagostomum*; lane 8, *Salmonella*; lane 9, *P. mirabilis*; lane 10, *S. aureus*; lane 11, negative control.

The optimal annealing temperature for the gene-specific PCR targeting *E. coli stx2* was 57 °C (Figure 3A); the optimal primer concentration was 0.40 μM (Figure 3B); and the optimal Mg^2+^ concentration was 1.5 mM (Figure 3C). The final established PCR reaction system consisted of 1.0 μL of template DNA, 0.125 μL of *Ex Taq*, 2.5 μL of 10 × *Ex Taq* Buffer (Mg^2+^ free), 4.0 μL of MgCl_2_ (25 mM), 2 μL of dNTP (2.5 mM each), and 0.8 μL each of the upstream and downstream primers (10 μM) for *E. coli stx2*, with the remaining volume made up of ddH_2_O. The annealing temperature was set at 57 °C, and the other reaction conditions were the same as those described for the gene-specific PCR targeting *E. coli eaeA*.

The original concentration of the positive plasmid pMD19-T-*stx2* extracted in this study was 1.82 × 10^9^ copies/μL. The sensitivity test showed that the detection limit of the gene-specific PCR targeting *E. coli stx2* was 182 copies (Figure 3D). The specificity test demonstrated that this PCR method amplified the expected band of 950 bp for *E. coli stx2*, while no amplification was observed for DNA from other common enteric microorganisms (Figure 3E).

#### 3.1.4. Optimal Reaction Conditions and Reaction System for the Gene-Specific PCR Targeting *C. perfringens cpa*

**Figure 4:**
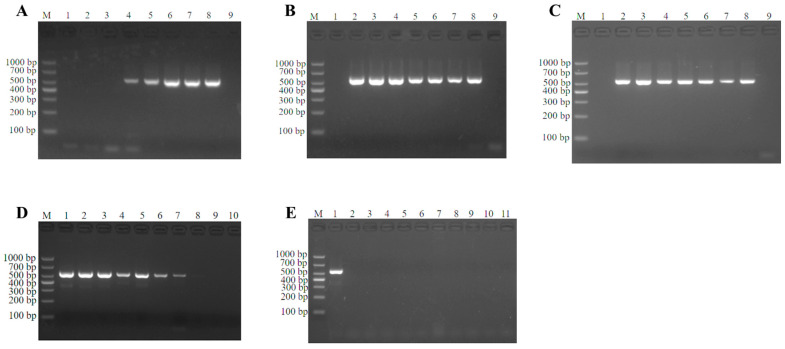
Establishment of the specific PCR assay for *C. perfringens cpa*. (**A**) Optimization of annealing temperature: M, DL1000 Marker; lanes 1–8, annealing temperature of 53.0 °C, 54.0 °C, 55.0 °C, 56.0 °C, 57.0 °C, 58.0 °C, 59.0 °C and 60.0 °C, respectively; lane 9, negative control. (**B**) Optimization of primer concentration: M, DL1000 Marker; lanes 1–8, primer concentrations of 0.16 μM, 0.24 μM, 0.32 μM, 0.40 μM, 0.48 μM, 0.56 μM, 0.64 μM, and 0.72 μM, respectively; lane 9, negative control. (**C**) Optimization of MgCl_2_ concentration: M, DL1000 Marker; lanes 1–8, MgCl_2_ concentrations of 0.5 mM, 1.0 mM, 1.5 mM, 2.0 mM, 2.5 mM, 3.0 mM, 3.5 mM, and 4.0 mM, respectively; lane 9, negative control. (**D**) Sensitivity test: M, DL2000 DNA Marker; lane 1, 1.99 × 10^9^ copies; lane 2, 1.99 × 10^8^ copies; lane 3, 1.99 × 10^7^ copies; lane 4, 1.99 × 10^6^ copies; lane 5, 1.99 × 10^5^ copies; lane 6, 1.99 × 10^4^ copies; lane 7, 1.99 × 10^3^ copies; lane 8, 1.99 × 10^2^ copies; lane 9, 1.99 × 10^1^ copies; lane 10, negative control. (**E**) Specificity test: M, DNA Marker DL1000; lane 1, *C. perfringens*; lane 2, *G. duodenalis*; lane 3, *Eimeria*; lane 4, *Moniezia*; lane 5, *E. bieneusi*; lane 6, *H. contortus*; lane 7, *Oesophagostomum*; lane 8, *Salmonella*; lane 9, *P. mirabilis*; lane 10, *S. aureus*; lane 11, negative control.

The optimal annealing temperature for the gene-specific PCR targeting *C. perfringens cpa* was 60 °C (Figure 4A); the optimal primer concentration was 0.32 μM (Figure 4B); and the optimal Mg^2+^ concentration was 1.5 mM (Figure 4C). The final established PCR reaction system consisted of 1.0 μL of template DNA, 0.125 μL of *Ex Taq*, 2.5 μL of 10 × *Ex Taq* Buffer (Mg^2+^ free), 4.0 μL of MgCl_2_ (25 mM), 2 μL of dNTP (2.5 mM each), and 0.8 μL each of the upstream and downstream primers (10 μM) for *C. perfringens cpa*, with the remaining volume made up of ddH_2_O. The annealing temperature was set at 60 °C, and the other reaction conditions were the same as those described for the gene-specific PCR targeting *E. coli eaeA*.

The original concentration of the positive plasmid pMD19-T-*cpa* extracted in this study was 1.99 × 10^9^ copies/μL. The sensitivity test showed that the detection limit of the gene-specific PCR targeting *C. perfringens cpa* was 199 copies (Figure 4D). The specificity test demonstrated that this PCR method amplified the expected band of 550 bp for *C. perfringens cpa*, while no amplification was observed for DNA from other common enteric microorganisms (Figure 4E).

#### 3.1.5. Optimal Reaction Conditions and Reaction System for the Gene-Specific PCR Targeting *Cryptosporidium 18S rRNA*

**Figure 5:**
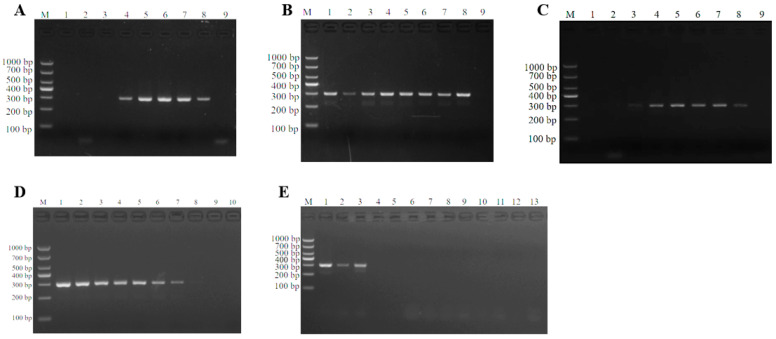
Establishment of the specific PCR assay targeting *Cryptosporidium 18S rRNA*. (**A**) Optimization of annealing temperature: M, DL1000 Marker; lanes 1–8, annealing temperature of 53.0 °C, 54.0 °C, 55.0 °C, 56.0 °C, 57.0 °C, 58.0 °C, 59.0 °C and 60.0 °C, respectively; lane 9, negative control. (**B**) Optimization of primer concentration: M, DL1000 Marker; lanes 1–8, primer concentrations of 0.16 μM, 0.24 μM, 0.32 μM, 0.40 μM, 0.48 μM, 0.56 μM, 0.64 μM, and 0.72 μM, respectively; lane 9, negative control. (**C**) Optimization of MgCl_2_ concentration: M, DL1000 Marker; lanes 1–8, MgCl_2_ concentrations of 0.5 mM, 1.0 mM, 1.5 mM, 2.0 mM, 2.5 mM, 3.0 mM, 3.5 mM, and 4.0 mM, respectively; lane 9, negative control. (**D**) Sensitivity test: M, DL2000 DNA Marker; lane 1, 9.74 × 10^8^ copies; lane 2, 9.74 × 10^7^ copies; lane 3, 9.74 × 10^6^ copies; lane 4, 9.74 × 10^5^ copies; lane 5, 9.74 × 10^4^ copies; lane 6, 9.74 × 10^3^ copies; lane 7, 9.74 × 10^2^ copies; lane 8, 9.74 × 10^1^ copies; lane 9, 9.74 copies; lane 10, negative control. (**E**) Specificity test: M, DNA Marker DL1000; lane 1, *C. parvum*; lane 2, *C. bovis*; lane 3, *C. ryanae*; lane 4, *G. duodenalis*; lane 5, *Eimeria*; lane 6, *Moniezia*; lane 7, *E. bieneusi*; lane 8, *H. contortus*; lane 9, *Oesophagostomum*; lane 10, *Salmonella*; lane 11, *P. mirabilis*; lane 12, *S. aureus*; lane 13, negative control.

The optimal annealing temperature for the gene-specific PCR targeting *Cryptosporidium 18S rRNA* was 58 °C (Figure 5A); the optimal primer concentration was 0.72 μM (Figure 5B); and the optimal Mg^2+^ concentration was 2.5 mM (Figure 5C). The final established PCR reaction system consisted of 1.0 μL of template DNA, 0.125 μL of *Ex Taq*, 2.5 μL of 10 × *Ex Taq* Buffer (Mg^2+^ free), 2.5 μL of MgCl_2_ (25 mM), 2 μL of dNTP (2.5 mM each), and 1.8 μL each of the upstream and downstream primers (10 μM) for the 18S rRNA gene of *Cryptosporidium*, with the remaining volume made up of ddH_2_O. The annealing temperature was set at 58 °C, and other reaction conditions were the same as those described for the gene-specific PCR targeting *E. coli eaeA*.

The original concentration of the positive plasmid pMD19-T-*18S rRNA* extracted in this study was 9.74 × 10^8^ copies/μL. The sensitivity test showed that the detection limit of the gene-specific PCR targeting *Cryptosporidium 18S rRNA* was 974 copies (Figure 5D). The specificity test demonstrated that this PCR method amplified the expected band of 300 bp for *C. parvum*, *C. bovis* and *C. ryanae*, while no amplification was observed for DNA from other common enteric microorganisms (Figure 5E).

### 3.2. Establishment of Multiplex PCRs

#### 3.2.1. Optimization of Multiplex PCR

**Figure 6:**
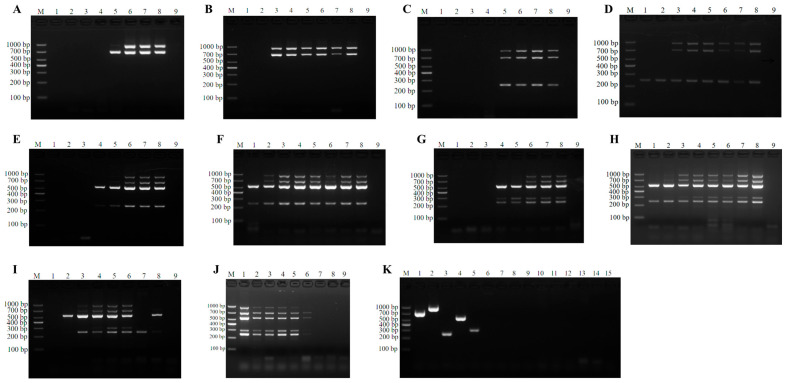
Establishment of the multiplex PCR assay. (**A**) Optimization of annealing temperature for the multiplex PCR targeting the *stx1* and *stx2* of *E. coli*: M, DL1000 Marker; lanes 1–8, annealing temperature of 53.0 °C, 54.0 °C, 55.0 °C, 56.0 °C, 57.0 °C, 58.0 °C, 59.0 °C and 60.0 °C, respectively; lane 9, negative control. (**B**) Optimization of primer concentration for the multiplex PCR targeting the *stx1* and *stx2* of *E. coli*: M, DL1000 Marker; lanes 1–8, primer concentrations of 0.16 μM, 0.24 μM, 0.32 μM, 0.40 μM, 0.48 μM, 0.56 μM, 0.64 μM, and 0.72 μM, respectively; lane 9, negative control. (**C**) Optimization of annealing temperature for the multiplex PCR targeting the *stx1*, *stx2*, and *eaeA* of *E. coli*: M, DL1000 Marker; lanes 1–8, annealing temperature of 53.0 °C, 54.0 °C, 55.0 °C, 56.0 °C, 57.0 °C, 58.0 °C, 59.0 °C and 60.0 °C, respectively; lane 9, negative control. (**D**) Optimization of primer concentration for the multiplex PCR targeting the *stx1*, *stx2* and *eaeA* of *E. coli*: M, DL1000 Marker; lanes 1–8, primer concentrations of 0.16 μM, 0.24 μM, 0.32 μM, 0.40 μM, 0.48 μM, 0.56 μM, 0.64 μM, and 0.72 μM, respectively; lane 9, negative control. (**E**) Optimization of annealing temperature for the multiplex PCR targeting the *stx1*, *stx2*, *eaeA* and *cpas*: M, DL1000 Marker; lanes 1–8, annealing temperature of 53.0 °C, 54.0 °C, 55.0 °C, 56.0 °C, 57.0 °C, 58.0 °C, 59.0 °C and 60.0 °C, respectively; lane 9, negative control. (**F**) Optimization of primer concentration for the multiplex PCR targeting the *stx1*, *stx2*, *eaeA* and *cpa*: M, DL1000 Marker; lanes 1–8, primer concentrations of 0.16 μM, 0.24 μM, 0.32 μM, 0.40 μM, 0.48 μM, 0.56 μM, 0.64 μM, and 0.72 μM, respectively; lane 9, negative control. (**G**) Optimization of annealing temperature for the multiplex PCR targeting the *stx1*, *stx2*, *eaeA*, *cpa* and *18S rRNA*: M, DL1000 Marker; lanes 1–8, annealing temperature of 53.0 °C, 54.0 °C, 55.0 °C, 56.0 °C, 57.0 °C, 58.0 °C, 59.0 °C and 60.0 °C, respectively; lane 9, negative control. (**H**) Optimization of primer concentration for the multiplex PCR targeting the *stx1*, *stx2*, *eaeA*, *cpa* and *18S rRNA*: M, DL1000 Marker; lanes 1–8, primer concentrations of 0.16 μM, 0.24 μM, 0.32 μM, 0.40 μM, 0.48 μM, 0.56 μM, 0.64 μM, and 0.72 μM, respectively; lane 9, negative control. (**I**) Optimization of MgCl_2_ concentration for the multiplex PCR targeting the *stx1*, *stx2*, *eaeA*, *cpa* and *18S rRNA*: M, DL1000 Marker; lanes 1–8, MgCl_2_ concentrations of 0.5 mM, 1.0 mM, 1.5 mM, 2.0 mM, 2.5 mM, 3.0 mM, 3.5 mM, and 4.0 mM, respectively; lane 9, negative control. (**J**) Sensitivity test of the multiplex PCR: M, DNA Marker DL1000; lanes 1–8, recombinant plasmid pMD19-T-*stx1* at concentrations ranging from 2.06 × 10^8^ copies/μL to 20.6 copies/μL, recombinant plasmid pMD19-T-*stx2* at concentrations ranging from 1.82 × 10^8^ copies/μL to 18.2 copies/μL, recombinant plasmid pMD19-T-*eaeA* at concentrations ranging from 1.30 × 10^8^ copies/μL to 13.0 copies/μL, recombinant plasmid pMD19-T-*cpa* at concentrations ranging from 1.99 × 10^8^ copies/μL to 19.9 copies/μL, and recombinant plasmid pMD19-T-*18S rRNA* at concentrations ranging from 9.74 × 10^7^ copies/μL to 9.74 copies/μL; lane 9, negative control. (**K**) Specificity test of the multiplex PCR: M, DNA Marker DL1000; lanes 1–3, DNA samples positive for *stx1*, *stx2*, and *eaeA* genes of *E. coli*; lane 4, *C. perfringens*; lane 5, *Cryptosporidium*; lane 6, *G. duodenalis*; lane 7, *Eimeria*; lane 8, *Moniezia*; lane 9, *E. bieneusi*; lane 10, *H. contortus*; lane 11, *Oesophagostomum*; lane 12, *Salmonella*; lane 13, *P. mirabilis*; lane 14, *S. aureus*; lane 15, negative control.

Based on the optimal reaction conditions and systems established for the single-gene-specific PCR targeting *E. coli stx1*, specific primers for *E. coli stx2* were first added to determine the optimal annealing temperature (60.0 °C; Figure 6A) and optimal primer concentration (0.32 μM; Figure 6B) for the multiplex PCR. Subsequently, specific primers for *E. coli eaeA* were added to determine the optimal annealing temperature (59.0 °C; Figure 6C) and optimal primer concentration (0.72 μM; Figure 6D). Following this, specific primers for *C. perfringens cpa* were added to determine the optimal annealing temperature (60.0 °C; Figure 6E) and optimal primer concentration (0.32 μM; Figure 6F). Finally, specific primers for *Cryptosporidium 18S rRNA* were added to determine the optimal annealing temperature (58 °C; Figure 6G) and optimal primer concentration (0.72 μM; Figure 6H). Subsequently, the Mg^2+^ concentration for the multiplex PCR was optimized, and the optimal Mg^2+^ concentration was determined to be 2.5 mM (Figure 6I).

The final established multiplex PCR reaction system consisted of 1.0 μL of template DNA, 0.125 μL of *Ex Taq* polymerase, 2.5 μL of 10 × *Ex Taq* Buffer (Mg^2+^ free), 2.5 μL of MgCl_2_ (25 mM), and 2.0 μL of dNTP (2.5 mM each), along with 0.8 μL of each specific primer (10 μM) for *C. perfringens cpa*, 1.0 μL for *E. coli stx1*, 0.8 μL for *E. coli stx2*, 1.8 μL for *E. coli eaeA*, and 1.8 μL for *Cryptosporidium 18S rRNA*. The remaining volume was made up of ddH_2_O for a total reaction volume of 25 μL. The annealing temperature was set at 58 °C, and the other reaction conditions were the same as those described for the gene-specific PCR targeting *E. coli eaeA*.

#### 3.2.2. Sensitivity Test of Multiplex PCR

In this study, the initial concentrations of the positive plasmids pMD19-T-*stx1*, pMD19-T-*stx2*, pMD19-T-*eaeA*, pMD19-T-*cpa* and pMD19-T-*18S rRNA* were 2.06 × 10^8^ copies/μL, 1.82 × 10^8^ copies/μL, 1.30 × 10^8^ copies/μL, 1.99 × 10^8^ copies/μL and 9.74 × 10^7^ copies/μL, respectively. Each plasmid DNA template was subjected to tenfold serial dilution and detected using the multiplex PCR assay established in this study. The minimum detectable plasmid copy numbers for the multiplex PCR were determined to be 2060 copies for *stx1*, 18200 copies for *stx2*, 1300 copies for *eaeA*, 1990 copies for *cpa*, and 974 copies for *18S rRNA* (Figure 6J).

#### 3.2.3. Specificity Test of Multiplex PCR

The multiplex PCR successfully amplified the target bands for *stx1* (706 bp), *stx2* (950 bp), *eaeA* (248 bp), *cpa* (550 bp) and *18S rRNA* (300 bp), while no amplification bands were observed for other common enteric pathogens (Figure 6K).

#### 3.2.4. Application of Multiplex PCR in Clinical Sample Test

**Figure 7:**
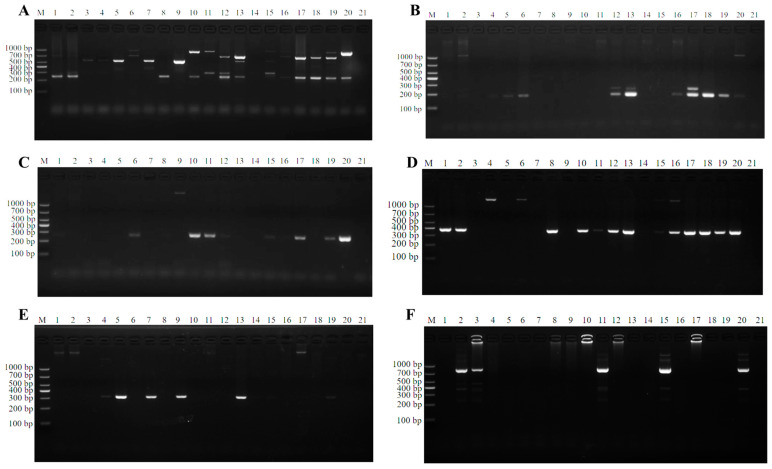
Detection of clinical samples. (**A**) Detection of clinical samples by multiplex PCR: M, DNA Marker DL2000; lanes 1–20, clinical samples; lane 21, negative control. (**B**) Detection of the *stx1* gene of *E. coli* in clinical samples by PCR: M, DNA Marker DL2000; lanes 1–20, clinical samples; lane 21, negative control. (**C**) Detection of *E. coli stx2* in clinical samples by PCR: M, DNA Marker DL2000; lanes 1–20, clinical samples; lane 21, negative control. (**D**) Detection of *E. coli eaeA* in clinical samples by PCR: M, DNA Marker DL2000; lanes 1–20, clinical samples; lane 21, negative control. (**E**) Detection of *C. perfringens cpa* in clinical samples by PCR: M, DNA Marker DL2000; lanes 1–20, clinical samples; lane 21, negative control. (**F**) Detection of *Cryptosporidium 18S rRNA* in clinical samples by PCR: M, DNA Marker DL2000; lanes 1–20, clinical samples; lane 21, negative control.

The final optimized multiplex PCR assay was applied to detect 20 faecal samples collected from clinically diarrheic calves on large-scale farms in Lingwu, Ningxia. The detection rates of the multiplex PCR for *stx1*, *stx2*, *eaeA*, *cpa* and *18S rRNA* were 55% (11/20), 50% (10/20), 60% (12/20), 45% (9/20) and 25% (5/20), respectively (Figure 7A). These rates were not significantly lower than those of the conventional PCR targeting *stx1* (60%, 12/20) (χ^2^ = 0.1023, *df* = 1, *p* = 0.7491) and *eaeA* (65%, 13/20) (χ^2^ = 0.1067, *df* = 1, *p* = 0.7440) (Figure 7B,C), nor significantly higher than those of the conventional PCR for *stx2* (45%, 9/20) (χ^2^ = 0.1003, *df* = 1, *p* = 0.7515) and *cpa* (40%, 8/20) (χ^2^ = 0.1023, *df* = 1, *p* = 0.7491) (Figure 7D,E), and were consistent with that of nested PCR for *18S rRNA* (25%, 5/20) (Figure 7F). To eliminate false positives, PCR products that were positive only by multiplex PCR were recovered from the gel and sent for sequencing, and the results confirmed that the two additional positive samples for *stx2* and *cpa* were true positives.

## 4. Discussion

*Escherichia coli*, *Clostridium perfringens* and *Cryptosporidium* are common pathogens associated with diarrhea in dairy calves [1]. These pathogens disrupt the intestinal immune barrier of the host by producing toxins or colonizing the intestine, causing substantial economic losses to the dairy farming industry [20,21,22]. Moreover, these pathogens have zoonotic potential, endangering human health. In the present study, we established a multiplex PCR assay for the simultaneous detection of *E. coli* virulence genes, *C. perfringens cpa* and *Cryptosporidium 18S rRNA* in dairy calves, which could provide an alternative approach for the rapid and effective detection of these selected virulence genes and *Cryptosporidium* in dairy calves.

Several diagnostic methods have been applied in the detection of pathogens associated with diarrhea in calves, including clinical history, physical examination, laboratory diagnosis, hematological and serum biochemical analyses, and postmortem examination [23]. Previous reports have suggested an association between age and infection with *E. coli*, *C. perfringens* and *Cryptosporidium*, but these methods lack specificity [6,24,25]. Laboratory diagnosis is widely used in the detection of diarrheic pathogens and encompasses multiple techniques, such as morphological examination (e.g., bacterial culturing and faecal flotation), immunological assays (e.g., ELISA and ICG), molecular-based assays (e.g., PCR and qPCR) and nanotechnology-based assays [23]. Morphological examination is considered the gold standard for pathogen detection, but it is time-consuming, lacks sensitivity and specificity, and requires well-trained researchers [26,27]. Immunological assays are fast and ideal for screening large numbers of samples, and can be applied to detect antibodies and antigens of diarrheic pathogens, but they lack specificity and cannot distinguish between previous and current infections [28]. Molecular-based assays exhibit high specificity and sensitivity when performed under appropriate operating procedures, and have been widely used in practice for the detection of *E. coli*, *C. perfringens* and *Cryptosporidium* [29].

To overcome the limitation of traditional PCR, which can only detect a single pathogen, multiplex PCR capable of simultaneously identifying multiplex infections has become increasingly popular [30]. Recently, several multiplex PCR assays have been developed for the detection of diarrheic pathogens. For example, Abed and Menshawy established a multiplex PCR for the detection of *E. coli* associated with diarrhea in calves, and identified the presence of EAEC and EHEC [31]. Pansri and colleagues developed a new multiplex quantitative PCR (qPCR) test, termed Enterit4Calves, which can detect and quantify pathogens responsible for diarrhea in calves, including *C. perfringens*, *E. coli* F5, bovine rotavirus, bovine coronavirus, *C. parvum* and *Eimeria* [32]. However, these methods typically focus on a limited set of selected pathogens, which restricts their application in clinical detection due to the regional diversity of pathogens.

A previous study in our laboratory identified a high prevalence of *E. coli* virulence genes (*stx1*, *stx2* and *eaeA*), *C. perfringens cpa*, and *Cryptosporidium* in diarrheic dairy calves in Ningxia Hui Autonomous Region, China [16]. Although a multiplex PCR has been developed to detect *E. coli* associated with diarrhea in calves, and another multiplex PCR has been established to detect *C. perfringens*, *E. coli* F5, bovine rotavirus, bovine coronavirus, *C. parvum* and *Eimeria* [31,32], neither method can simultaneously detect *E. coli* virulence genes (*stx1*, *stx2* and *eaeA*), *C. perfringens cpa* and *Cryptosporidium*. To address this limitation, the present study aimed to establish a multiplex PCR for the simultaneous detection of the selected *E. coli* virulence genes, *C. perfringens cpa* and *Cryptosporidium*, thereby facilitating a better understanding of the prevalence of these virulence genes and pathogens in Ningxia and contributing to the prevention and control of calf diarrhea in this region. In this study, gene-specific PCR assays were first established for *E. coli stx1*, *stx2* and *eaeA*, *C. perfringens cpa*, and *Cryptosporidium 18S rRNA*. Subsequently, based on the optimized PCR assay targeting *stx1*, specific primers for *stx2*, *eaeA*, *cpa* and *18S rRNA* were sequentially added to develop a multiplex PCR assay for the simultaneous detection of the selected targets. When applied to 20 clinical samples from diarrheic calves, the established multiplex PCR assay showed detection rates for *E. coli stx1* and *eaeA* that were slightly lower than those obtained with conventional PCR assays. This may be attributed to the presence of multiple primer pairs in the multiplex PCR system, which can lead to primer–dimer formation or preferential amplification of other targets, thereby reducing the amplification efficiency of *stx1* and *eaeA* and resulting in lower detection rates [23,32]. Conversely, two samples that were positive for *stx2* and *cpa* in the multiplex PCR were negative in the conventional single PCR assays, indicating that although conventional wisdom suggests that singleplex PCR is more sensitive than multiplex PCR, some positive samples may be detected only by multiplex PCR. Similar findings have been reported previously [33]. The detection rate for *Cryptosporidium 18S rRNA* was consistent with that obtained by nested PCR.

Notably, the multiplex PCR assay is approximately 10–100 times less sensitive for several targets. Several reasons may account for this phenomenon [34,35,36]. First, competition for and depletion of reaction components can occur. In a multiplex system, all reactions share limited reagents, as multiple primers and polymerases coexist in a single tube and compete for finite resources such as DNA polymerase, dNTPs, and magnesium ions. Second, there is a risk of non-specific interactions. The presence of a large number of oligonucleotides increases the chance of non-specific binding among them. Third, intrinsic physical limitations of the reaction system may also play a role. PCR requires balancing parameters such as annealing temperature, which may compromise amplification efficiency for certain targets. Therefore, future studies should focus on improving and validating the sensitivity of the multiplex PCR to enhance its practicality.

Although the established multiplex PCR assay could detect the selected *E. coli* virulence genes, *C. perfringens cpa* and *Cryptosporidium 18S rRNA* in these 20 dairy calf samples, several limitations may restrict its practical application and should be addressed in future studies. First, the present study lacked precise characterization and deposit of control strains, which may hinder independently replicating the experiment in another laboratory. Therefore, future studies should apply reference strains from international collections or public databases. Second, this study did not comply with WOAH guidelines for veterinary diagnostic tests for infectious diseases, as evidenced by the insufficient and unrepresentative clinical sample, lack of repeatability analysis, absence of external reproducibility assessment, and inadequate reaction control design. Third, the multiplex PCR requires validation on a substantially larger dataset, including samples from multiple farms and different geographical regions, comprising both positive and negative samples, along with statistical comparison against reference methods. Fourth, the multiplex PCR did not include bovine rotavirus, bovine coronavirus, bovine viral diarrhea virus, or other microorganisms. Given that calf diarrhea is a multifactorial disease, broader specificity testing incorporating more of the aforementioned microorganisms would strengthen assay validation in future studies. Fifth, independent gold standards were not included for comparison with the multiplex PCR, which may affect the assessment of the assay’s performance.

## 5. Conclusions

The multiplex PCR assay established in this study has the potential to simultaneously detect the selected three *E. coli* virulence genes, *C. perfringens cpa* and *Cryptosporidium 18S rRNA*. However, future studies are urgently needed to validate this assay using more samples from diverse geographical locations to assess its application prospects.

## Figures and Tables

**Table 1:** PCR primers for *E. coli* virulence genes, *C. perfringens cpa* and *Cryptosporidium 18S rRNA*.

Pathogen	Gene	Primer Name	Primer Sequence (5′–3′)	Fragment Size (bp)
*E. coli*	*eaeA*	F	TATGCTTAGTGCTGGTTTAGGA	248
		R	CCTTCATCATTTCGCTTTCA	
	*stx1*	F	ATTACAGACTATTTCATCAGGAGG	706
		R	CGGACACATAGAAGGAAACTCA	
	*stx2*	F	GGTTTTTCTTCGGTATCCTATTC	950
		R	CGCCATAAACATCTTCTTCATACT	
*C. perfringens*	*cpa*	F	TAGGTTCTACTTATCCAGATTATG	550
		R	GCTGTTCCTTTTTGAGAGTTAG	
*Cryptosporidium*	18S rRNA	F	TTTACTTTGAGAAAATTAGAGTGCTT	300
		R	CAATCTCTAGTTGGCATA	

## Data Availability

Data are contained within the article.

## Supplementary Materials

The following supporting information can be downloaded at: https://www.mdpi.com/article/10.3390/biology15120921/s1, Table S1: Sequences used in this study for plasmid construction.
