# Supplementary material for: Development of a Multiplex PCR Assay for Selected Escherichia coli Virulence Genes, Clostridium perfringens cpa and Cryptosporidium 18S rRNA in Faecal Samples from Diarrheic Dairy Calves
Source: Biology (Basel). 2026 Jun 12;15(12):921. doi: 10.3390/biology15120921 (PMC13295645; doi:10.3390/biology15120921)
Supplement: Supplementary file 1 [file biology-15-00921-s001.zip › biology-4361028-supplementary.pdf]

Table S1. Sequences used in this study for plasmid construction

Gene name	Sequence (5’-3’)
stx1	ATTACAGACTATTTCATCAGGAGGTACGTCTTTACTGATGATTGATAG<br>TGGCACAGGGGATAATTTGTTTGCAGTTGATGTCAGAGGGATAGATC<br>CAGAGGAAGGGCGGTTTAATAATCTACGGCTTATTGTTGAACGAAAT<br>AATTTATATGTGACAGGATTTGTTAACAGGACAAATAATGTTTTTTAT<br>CGCTTTGCTGATTTTTCACATGTTACCTTTCCAGGTACAACAGCGGTT<br>ACATTGTCTGGTGACAGTAGCTATACCACGTTACAGCGTGTTGCAGG<br>GATCAGTCGTACGGGGATGCAGATAAATCGCCATTCGTTGACTACTT<br>CTTATCTGGATTTAATGTCGCATAGTGGAACCTCACTGACGCAGTCTG<br>TGGCAAGAGCGATGTTACGGTTTGTTACTGTGACAGCTGAAGCTTTA<br>CGTTTTCGGCAAATACAGAGGGGATTTCGTACAACACTGGATGATCT<br>CAGTGGGCGTTCTTATGTAATGACTGCTGAAGATGTTGATCTTACATT<br>GAACTGGGGAAGGTTGAGTAGTGTCCTGCCTGATTATCATGGACAAG<br>ACTCTGTTCGTGTAGGAAGAATTTCTTTTGGAAGCATTAATGCAATTC<br>TGGGAAGCGTGGCATTAATACTGAATTGTCATCATCATGCATCGCGA<br>GTTGCCAGAATGGCATCTGATGAGTTTCCTTCTATGTGTCCG
stx2	GGTTTTTCTTCGGTATCCTATTCCCGGGAATTTACGATAGACTTTTCG<br>ACTCAACAAAGTTATGTATCTTCGTTAAATAGTATACGGACAGAGATA<br>TCGACCCCTCTTGAACATATATTTCAGGGGACCACATCGGTGTCTGTT<br>ATTAACCACACCCCACCGGGCAGTTATTTTGCTGTGGATATACGAGG<br>GCTTGATGTCTATCAGGCGCGTTTTGACCATCTTCGTCTGATTATTGA<br>GCAAAATAATTTATATGTGGCCGGGTTCGTTAATACGGCAACAAATAC<br>TTTCTACCGTTTTTCAGATTTTACACATATATCAGTGCCCGGTGTGAC<br>AACGGTTTCCATGACAACGGACAGCAGTTATACCACTCTGCAACGTG<br>TCGCAGCGCTGGAACGTTCCGGAATGCAAATCAGTCGTCACTCACT<br>GGTTTCATCATATCTGGCGTTAATGGAGTTCAGTGGTAATACAATGAC<br>CAGAGATGCATCCAGAGCAGTTCTGCGTTTTGTCACTGTCACAGCAG<br>AAGCCTTACGCTTCAGGCAGATACAGAGAGAATTTCGTCAGGCACT<br>GTCTGAAACTGCTCCTGTGTATACGATGACGCCGGGAGACGTGGAC<br>CTCACTCTGAACTGGGGGCGAATCAGCAATGTACTTCCGGAGTATCG<br>GGGAGAGGATGGTGTCAGAGTGGGGAGAATATCCTTTAATAATATAT<br>CGGCGATACTGGGCACTGTGGCCGTTATACTGAATTGTCATCATCAG<br>GGGGCGCGTTCTGTTCGCGCCGTGAATGAAGATAGTCAACCAGAAT<br>GTCAGATAACTGGCGACAGGCCCGTTATAAGAATAAACAATACATTA<br>TGGGAAAGTAATACAGCTGCAGCGTTTCTGAACAGAAAGTCACAGT<br>TTTTATATACAACGGGTAAATAAAGGAGTTAAGTATGAAGAAGATGT<br>TTATGGCG
eaeA	TATGCTTAGTGCTGGTTTAGGATTGTTTTTTATGTTAACCAGAACTC<br>ATTTGCAAACGGTGAAAATTATTTTAAATTGAGTTCAGATTCAAAAC<br>TGTTAACTCAAAATGTTGCTCAGGATCGCCTTTTTTATACGTTGAAAA<br>CAGGTGAAACTGTTTCCAGTATTTCTAAATCACAAGGTATCAGTTTAT<br>CCGTAATTTGGTCACTGAATAAACATTTATACAGTTCTGAAAGCGAA<br>ATGATGAAGG

cpa	TAGGTTCTACTTATCCAGATTATGATAAGAACGCCTATGTTCTATATCAAGATCATTTCTGGGATCCTGATACAGATAATAATTTCTCAAAGGATAATAGTTGGTATTTAGCTTATTCTATACCTGACACAGGGGAATCACAAATAAGAAAATTTTCAGCATTAGCTAGATATGAATGGCAAAGAGGAAACTATAAACAAGCTACATTCTATCTTGGAGAGGCTATGCACTATTTTGGAGATATAGATACTCCATATCATCCTGCTAATGTTACTGCCGTTGATAGCGCAGGACATGTTAAGTTTGAAACTTTTGCAGAGGAAAGAAAAGAACAGTATAAAATAAACACAGCAGGTTGCAAAACTAATGAGGATTTTTATGCTGATATCTTAAAAAACAAGGATTTTAATGCATGGTCAAAAGAATATGCAAGAGGTTTTGCTAAAACAGGAAAATCAATATACTATAGTCATGCTAGCATGAGTCATAGTTGGGATGATTGGGACTATGCAGCAAAGGTAACTTTAGCTAACTCTCAAAAAGGAACAGC
18S rRNA	TTTACTTTGAGAAAATTAGAGTGCTTAAAGCAGGCTATTGCCTTGAATACTCCAGCATGGAATAATATTAAGGATTTTTATTCTTCTTATTGGTTCTAGAATAAAAATGATGATTAATAGGGACAGTTGGGGGCATTTGTATTTAACAGTCAGAGGTGAAATCCTTAGATTTGTTAAAGACAAACTACTGCGAAAGCATTTGCCAAGGATGTTTTCATTAATCAAGAACGAAAGTTAGGGGATCGAAGACGATCAGATACCGTCGTAGTCTTAACCATAAACTATGCCAACTAGAGATTG
